# Holding space: a participatory exploration of first nations health professionals’ experiences supporting cancer patients through hospital-based treatment

**DOI:** 10.1007/s00520-025-09524-4

**Published:** 2025-05-21

**Authors:** Kate Anderson, Nicole Hewlett, Elaina Elder-Robinson, Gail Garvey, Rebecca Murray, Bonnie Chatfield, Lisa Fletcher, Catherine Noble, Kirsten Howard

**Affiliations:** 1 Yardhura Walani Centre, Australian National University, Canberra, Australia; 2 School of Public Health, University of Queensland, Sta. Lucia, QLD Australia; 3 South West Sydney Local Health District, NSW Health, St. Leonards, NSW Australia; 4 Cancer Centre Eurobodalla, NSW Health, St. Leonards, NSW Australia; 5 North Coast Local Health District, NSW Health, St. Leonards, NSW Australia; 6 Dubbo Health Service, NSW Health, St. Leonards, NSW Australia; 7 Leeder Centre for Health Policy, Economics and Data, University of Sydney, Camperdown, NSW Australia; 8 Centre of Alcohol Policy Research, La Trobe University, Melbourne, Australia

**Keywords:** First Nations, Healthcare, Cancer care

## Abstract

**Background:**

Colonial mechanisms continue to inflict trauma on Aboriginal and Torres Strait Islander peoples, the First Nations peoples of Australia. Consequence of this trauma is a disproportionately high rate of cancer mortality experienced among First Nations peoples and inequities in access to cancer services that are culturally responsive. There is a critical need for cancer care that supports First Nations peoples’ holistic health and wellbeing. Engagement with First Nations health staff is a known element of culturally safe healthcare, however the experiences of and challenges facing First Nations staff working in cancer care are unclear.

**Method:**

Conducted as part of the What Matters to Adults Implementation (WM2A-Implementation) study, this paper presents the findings of a participatory approach to explore the experiences of four First Nations Health Professionals (FNHPs) providing holistic cancer care for First Nations peoples within cancer services located in public hospitals. Ten Yarning Circles were conducted by a First Nations researcher over a 12-month period. All were transcribed and a Knowledge Synthesis method employed a reflexive thematic analysis approach.

Meaning making.

FNHPs shared their experiences of working in a complex, highly pressured, and sometimes adverse space. FNHPs worked to support and advocate for their patients, create culturally safe spaces, and support and guide colleagues to the provision of culturally safe, patient-centred cancer care. Our knowledge synthesis revealed six intersecting themes that encapsulate their experiences: *holding space; advocacy for patients; incorporating First Nations ways; serving your community; being everything to everyone;* and the *stigma of the role*.

**Discussion:**

These findings have implications for guiding cancer services to create an environment where First Nations staff are respected and given adequate resources, space, and support to deliver culturally grounded and supportive care to First Nations patients and their families. Specifically, services need to recognise the value of FNHPs in patient-centred care; balance this value with the burden on FNHPs; foster greater inclusion of First Nations culture and knowledges in mainstream healthcare; and actively focus on reducing racism and stigma facing FNHPs.

## Introduction

Before the British invasion and colonisation of Australia, Aboriginal and Torres Strait Islander peoples (hereafter respectfully referred to as First Nations peoples) thrived physically, mentally, and spiritually for millennia. [1] Ongoing colonial processes continue to substantially impact the health and well-being of First Nations peoples, resulting in lower life expectancies than their non-First Nations counterparts. [2, 3] Cancer is now the leading cause of death for First Nations peoples in Australia, and the gap in cancer mortality between First Nations and non-First Nations Australians is widening. [2] The widening cancer mortality gap between First Nations and non-First Nations Australians is rooted in the legacies of colonisation. Such legacies are evident in the numerous barriers First Nations peoples experience at every point on the cancer care continuum. [4] First Nations peoples experience low cancer screening rates; [4] late cancer diagnosis; [5, 6] and high rates of comorbid illness. [6] Contributing factors include: high levels of reported racism; [7] validated mistrust of medical institutions; [8, 9] urban-centric treatment facilities; [4, 10] language and cultural differences from care providers; [4–6, 8, 11] and substantial economic, health and social disadvantages [12].

There is a critical need to provide cancer care that is both clinically effective and culturally inclusive, respecting the diverse and holistic well-being of First Nations peoples [7, 13, 14]. First Nations health staff are a critical element of culturally safe healthcare, as they bring culturally-informed knowledge, connection and skill to service delivery [15]. The presence of First Nations staff is an embedded element of the *Cultural safety in health care for Indigenous Australians: monitoring framework. *[16] However, First Nations peoples are significantly underrepresented in the health workforce, which suggests many First Nations peoples may have unmet health, cultural, emotional and social needs when accessing cancer care [17].

There is increasing attention on building representation of First Nations peoples in the health care workforce [17–20]. The recently published Australian Cancer Plan emphasises the importance of culturally safe care and the role of First Nations health professionals in achieving better cancer outcomes for First Nations communities [19]. Furthermore, the Aboriginal and Torres Strait Islander Cancer Plan identifies the need to engage, recruit and retain a strong, skilled First Nations health workforce, to prioritise leadership through First Nations peoples and support their career development, and to strengthen meaningful career progression pathways for First Nations peoples [20]. Despite this there are well documented barriers in achieving this, for example, First Nations health workers experience the compounding impacts of racism, cultural load, feeling undervalued, limited career progression, and low remuneration [15, 21–23]. The hegemony of Western practices in medical settings means that First Nations cultural knowledge is often not valued, leading to First Nations health staff feeling excluded and devalued [24]. Similarly, the insidious systemic and personal racism experienced by First Nations health staff contributes to high rates of burnout and exiting the profession [25]. Further, First Nations health staff experience increased cultural load, as they themselves are part of the local community whilst simultaneously providing care to the community through Westernised structures [15]. These elements are magnified in the cancer care setting, due to the complex technical, cultural and emotional demands on its workforce [26]. While emerging research is exploring patient navigator programs for First Nations peoples accessing cancer services [27], and successful collaborations between First Nations and non-First Nations cancer staff [28, 29], there is little published information about the experiences of First Nations staff working in a cancer care setting [24].

Given the central role that First Nations staff play in facilitating equitable access to culturally safe care and the adversity experienced in these roles, it is essential to understand the experiences of First Nations health professionals (FNHPs) working in cancer care. In this paper, we present the findings of a participatory, qualitative exploration of the experiences of four FNHPs providing holistic cancer care to First Nations Peoples in a public hospital setting over 12 months. This paper provides unique insights into the realities of working at the interface of mainstream cancer treatment services and First Nations communities as a FNHP. The implications for cancer services and systems are explored and recommendations for more effectively supporting the FNHPs in order to strengthen cancer care for First Nations peoples are provided.

## Methods

This paper presents Knowledge shared by the four FNHPs who were employed as field staff for the What Matters to Adults Implementation (WM2 A—implementation) research project. In this project, the research team partnered with the Cancer Institute NSW, a pillar organisation within NSW Health, providing the strategic direction for cancer control in the Australian state of New South Wales (NSW), to pilot the implementation of a newly developed wellbeing measure (the What Matters to Adults—WM2 A) measure,[30, 31]. WM2 A is a 32-item culturally grounded measure that captures holistic wellbeing across 10 dimensions: balance and control; hope and resilience; caring for others; culture and country; spirit and identity; feeling valued; connection with others; access; racism and worries; and pride and strength. WM2 A was implemented over a 12-month period in 2023–24 within eight cancer services across four local health districts (LHDs) in NSW.

This component of the project used a participatory approach to work in close collaboration with the FNHPs [32] to explore and understand their experiences of administering the WM2 A measure with First Nations peoples accessing NSW cancer services over a 12-month period. While the experiences of FNHPs were originally intended to be collected in their relevance to the efficacy of administering and implementing the measure, it quickly became apparent that the wellbeing of FNHPs was a critical aspect of the successful implementation of the measure. Given this situation, the research team added a monthly Yarn with FNHPs, facilitated by a First Nations researcher to create a culturally safe space, to ensure that these Knowledge Holders could share their experiences of working in mainstream cancer services to support the wellbeing of First Nations peoples with cancer in ways that would enable Knowledge Sharing to highlight the need for change.

### Indigenist methodology

In accordance with Rigney’s Indigenist research methodologies, this qualitative study prides itself in its act of resistance by contributing to First Nations peoples’ self-determination, privileging First Nations voices in the research, and upholding political integrity and alignment with First Nations liberatory values [33]. First Nations leadership drives the study, ensuring the research reflects community priorities and amplifies First Nations voices. By privileging First Nations knowledges, experiences, and agency this approach upholds cultural sovereignty and challenges dominant research paradigms, both of which are central to decolonising research processes. The research terminology used in this paper is intended to reinforce the positioning First Nations knowledge holders as expert researchers, including terms such as, shared knowledge, knowledge holders, knowledge creation, knowledge synthesis, meaning making, and knowledge sharing [34]. The project is First Nations co-led, and has engaged, employed, and trained First Nations researchers and health staff. The WM2 A Project Advisory Group was engaged throughout the WM2 A Project and was involved in guiding the development of the WM2 A-Implementation Project, ensuring all research processes were oriented towards First Nations ways of conducting research. The Cancer Institute NSW partnered with the research team for the WM2 A—implementation project, and their existing advisory and governance committees provided guidance and input for the project: the Aboriginal Cancer Advisory Group and NSW Aboriginal Cancer Strategic Partnerships and Performance Committee.

### Positionality

The project team is comprised of First Nations and non-First Nations people with varied lived experience in the health and research sector. As a team, we have worked collaboratively to orient our research towards, and prioritise and privilege, the voices, culture, strengths, and wellbeing of First Nations people. Each member within our team brings their own unique history and perspective, and we collectively acknowledge the importance of recognising this diversity and how it impacts our work [35].

KA is a non-First Nations Australian senior researcher experienced in conducting collaborative qualitative research with First Nations researchers and communities. NH is a proud Palawa researcher with experience in translating healing-informed approaches and supporting the wellbeing of Aboriginal and Torres Strait Islander peoples working in health care. EE is a non-First Nations health practitioner working in an Aboriginal Community Controlled Health Organisation, and junior researcher, with research experience in First Nations cancer and wellbeing. GG is a Kamilaroi woman and senior researcher with expertise in mixed methods research and is passionate about achieving health equity for First Nations peoples through a focus on cancer and well-being research; she co-leads this WM2 A-Implementation study. RM is a proud Kamilaroi woman and an Aboriginal Health Worker in Cancer, with a strong interest in supporting equity in health and wellbeing outcomes for our First Nations people. BC is a proud Wiradjuri women living in Yuin nation and is an endorsed enrolled nurse and healthcare worker. LF is a proud Ngurrubul woman with Kamilaroi ties and a registered nurse working as a First Nations liaison nurse supporting mob through their cancer treatments in Gumbaynggirr country. CN is a proud Wiradjuri woman, an Aboriginal Health Worker and Aboriginal Cancer Care Coordinator advocating for patients’ medical and cultural needs during their cancer journey. KH is a non-First Nations senior researcher experienced in conducting research with First Nations researchers and communities; she co-leads this WM2 A-Implementation study.

### Knowledge holders and knowledge creation

The WM2 A—Implementation project used a three-phase implementation-evaluation, mixed methods design informed by the i-PARIHS framework[36] to implement WM2 A into the Cancer Institute Patient Reported Measures system in eight cancer services across four LHDs. Four FNHPs were recruited, one in each LHD, employed in part-time roles for a 12-month period during the implementation phase of the project. These roles were responsible for administering the WM2 A measure to First Nations patients accessing cancer services, including treatment and providing holistic support for the wellbeing needs identified by the measure. The professional roles of the FNHPs were registered nurse, enrolled nurse, and aboriginal health workers. FNHPs were recruited and employed through partner organisations: the LHDs and cancer services.

During the 12-month implementation phase, monthly Yarning Circles with FNHPs were facilitated by a First Nations researcher (NH) experienced in supporting the wellbeing of First Nations workforce using Yarning Circles. ‘Yarning’ is a First Nations communication style that facilitates an exchange of knowledge and stories from a space of connection, trust and respect [37] In research, Yarning is a First Nations methodology that enables First Nations ways of sharing and understanding knowledges to co-create new knowledges to be privileged [37]. The purpose of the Yarning Circles for this project was firstly, to support the wellbeing of FNHPs implementing the measure and secondly, understand their experiences over the implementation period. A Yarning Guide was utilised to help guide the FNHP Yarning Circles alongside prompts from the Yarn facilitator (see Appendix 1). Each yarn included personal and professional experiences when implementing WM2 A.

### Ethics

Our research was conducted in accordance with the National Health and Medical Research Council’s (NHMRC) guidelines for conducting ethical research with Aboriginal and Torres Strait Islander peoples [38]. The six core values outlined in these NHMRC guidelines for research with First Nations Peoples are: spirit and integrity, cultural continuity, equity, reciprocity, respect, and responsibility [38]. The aims of embedding these values into research is to: respect the shared values of First Nations Peoples; is relevant for First Nations priorities, needs and aspirations; develops long-term ethical relationships among researchers, institutions and sponsors; and develops best practice ethical standards of research [38]. This study embodies all of the core values outlined by NHMRC to achieve these aims. Our research team is First Nations led, and majority First Nations comprised. FNHPs are included as co-researchers in all aspects of this research, including as Knowledge Holders, as participants in the Knowledge synthesis process and in the translation of this research in the written paper. The knowledge creation and knowledge synthesis sessions were structured to create a comfortable, accessible and safe space for FNHPs to share their truth. This approach purposely ensured that the priorities, needs and aspirations of FNHPs, who are the focus of this piece of research, were able to be collected and reflected in ways that are respectful and useful for guiding change in hospital settings.

Ethical approval was obtained from the Aboriginal Health and Medical Research Centre (1849/21) and the University of Sydney Human Ethics Research Committee (2021/710). Ethics ratification was given by the University of Queensland Human Ethics Research Committee (2022/HE000067). Site-specific ethical approval was obtained for Coastal Network, South-Western Sydney LHD, Mid North Coast Cancer Institute, Bathurst Health Service, Dubbo Base Hospital and Orange Health Service (2021/ETH12016). Informed consent was obtained from all Knowledge Holders included in the study.

#### Knowledge synthesis

Knowledge Synthesis of the FNHP Yarning Circles used an iterative and circular First Nations method called collaborative yarning methodology (CYM) [39]. Our research team has used CYM effectively in a number of studies associated with our broader body of work in First Nations wellbeing [40, 41]. CYM aims to privilege the voices of the First Nations peoples who have contributed to the research and is led by First Nations researchers. In the current study, the CYM process included the four FNHPs as research team members and knowledge holders. The Yarning Circles were audio-recorded, transcribed verbatim and uploaded into analysis software NVivo14 [42]. A reflexive thematic analysis [43] approach was used to support a process of knowledge synthesis. KA and EE reviewed Yarn transcripts to arrange the shared knowledge from FNHPs into broad themes. These themes, with supporting raw data, were collated and shared with the FNHP group by the Yarn facilitator over two sessions. FNHPs reviewed and revised the broad themes to further progress the knowledge synthesis and shape and refine the final themes.

## Results

Ten Yarning Circles were conducted with the four FNHPs, employed across four LHD sites and eight cancer services to administer the WM2 A measure to First Nations cancer patients and support the wellbeing needs of patients. Yarning Circles were held approximately monthly between March 2023 and March 2024. All Yarning Circles were facilitated by a First Nations researcher (NH) and lasted between 60 and 135 min. The Yarns followed a characteristic Research Yarn format with space and flexibility for Social Yarning and Research Yarning to coexist [37]. FNHPs shared their stories and experiences with the facilitator, while also offering guidance, affirmation and wellbeing support to each other.

### Meaning making

The knowledge synthesis of the Yarning Circles transcripts revealed that FNHPs’ experiences of supporting the wellbeing of First Nations cancer patients are complex and highly pressured, in both personal and professional capacities. FNHPs must balance the responsibility of supporting the diverse needs of patients and advocating for these needs to be met by a Western health care system, often with non-First Nations health professionals who have limited cultural competency. The findings detail these experiences and reveal how FNHPs navigate the conflicting elements of their workplace, whilst striving to maintain integrity in meeting their cultural responsibilities to their own communities. The following six overarching and intersecting themes were identified through applying CYM to the Yarning Circle transcripts:

1. Holding space
2. Patient Advocacy
3. Incorporating First Nations ways
4. Serving your community
5. Being everything to everyone
6. Stigma of the role

#### Holding space

FNHPs commonly described their role as *holding space* for patients within an unfamiliar and sometimes unwelcoming context of hospital cancer care. The ability to hold space was said to be underpinned by the development of a strong and trusting connection between the FNHP and the patient. The establishment of this type of meaningful connection took time to develop and foster. It was important that FNHPs demonstrated to patients that they were genuine, caring, trustworthy and culturally sensitive to the circumstances of the patient. Once formed, this connection allowed the FNHP to create and maintain a safe space for the patient to share their concerns and circumstances honestly and without judgement, which is a critical basis for being able to address patients’ holistic wellbeing needs.

*We’ve got to have that hold space. We definitely do because it can’t be just about rolling out the measure, having that space for a yarn and a yarn can’t just take five minutes. [FNHP 2, Yarn 10]*

*You would see the patient at least three or four times before you even do the survey or even consent to it … Because you know what mob’s like, they don’t trust nobody. Unless they’re good with you, you’re not going to get nothing from them. [FNHP 3, Yarn 7]*

*So, you’re working with these people, you allocate at least an hour with them when you first meet them, because you don’t want to just go, “Hey, bye, see you later,” because you’re not getting that connection. [FNHP 3, Yarn 6]*

Conversations between FNHPs and patients were often highly emotional, for both the patient and the FNHP. While these discussions sometimes occasioned sad and distressing emotions, FNHPs felt that giving patients the chance to share their stories and their realities with someone who understood and could provide some support, during their cancer treatment journey, gave patients strength and hope.

For FNHPs, the content of the WM2 A measure and Yarns with patients that accompanied its administration similarly brought up mixed emotions. FNHPs reflected that the content of Yarns could be triggering, in the sense that what the patient was going through, or their previous experiences, could be mirrored in the FNHPs own personal life and experiences. The opportunity for the patient to bond and feel comfortable sharing their story could create bidirectional comfort and healing.

*But it was just so nice to see the connection. Yeah. And it was just – I felt – I went home and said to my husband, “I had a really good day at work today.” [FNHP 4, Yarn 7]*

In addition, administering non-First Nations-specific patient-reported outcome measures with First Nations patients can be challenging and, at times, overwhelming. The language used to describe symptoms and distress, such as terms like “nausea” can be difficult for patients to understand. Additionally, these questions may place FNHPs in uncomfortable situations and, in some cases, may be culturally inappropriate, when it comes to Women’s Business and Men’s Business (First Nations cultural roles, customs and practices that are specific and sacred to females and males, respectively). Working closely with First Nations patients meant that FNHPs were often managing holistic patient issues. These were noted to range from racist interactions in a variety of settings, intergenerational traumas, and tough times within families. FNHPs spoke about how these interactions affected them personally, including prompting them to reflect on their own familial and community experiences, bringing up their own past traumas, or fuelling anger towards the injustice of Western systems and society.

*When you have a lot of trauma as well and you’re exposed to theirs, it kind of brings up stuff that you suppressed. So, then you have kind of unpack that at the same time. [FNHP 3, Yarn 1]*

#### Advocacy for patients

Throughout the Yarning Circles, FNHPs outlined their roles in guiding and advocating for patients. FNHPs saw themselves as a bridge between First Nations knowledges and Western medical settings. This bridging allowed FNHPs to be powerful advocates for their patients and to find solutions for a variety of issues and difficulties patients were facing.

*They know they’re missing something and they’re looking for it and you’re literally like the pathfinder and you’re bridging that there. [FNHP 3, Yarn 1]*

*What’s going on with them that they don’t want to talk about it over the phone? Why aren’t they answering the phone? There’s something else deeper going on than just their treatment or their appointment. Something else within the kinship of the family that’s going on. And I find our Western clinical staff don’t always get that. [FNHP 1, Yarn 1]*

*The doctor could see he wasn’t himself but wasn’t aware of the background stuff. So that’s where you can utilise your skillset to be able […] to share that knowledge with the specialist, with the consultants, with who needs it. [FNHP 4, Yarn 10]*

Sometimes advocacy is needed for specific instances due to inadequate and tone-deaf management of the patient within the health service. In these cases, FNHPs would go to great lengths to ensure that the patient’s concerns were addressed appropriately, which sometime involved direct petitions for action to aboriginal liaison officers (ALOs), medical specialists, and senior medical directors.

*I always validate them by saying, “I recognise that this is an incredibly distressing time for you.” So, whilst you’re in the midst of working through that, I on the other hand will be following up with the ALOs so that they can provide some extra supports. And if they want to complain, they can escalate it that way. [FNHP 3, Yarn 6]*

FNHPs described systematic barriers facing First Nations cancer patients in Western health services that prevented some clients from accessing care. They could see the difficulties that many patients face in attending their appointments, including home and family responsibilities, challenges with finance and transport, and the stigmatisation that some First Nations patients experienced from medical staff. FNHPs described hospital staff expressing ignorant and or unempathetic attitudes towards non-attendance by First Nations patients. FNHPs reported advocating for patients in such circumstances, which often resulted in personal distress for FNHPs.

*They’ll go, ‘Oh, they didn’t attend four appointments, they’re no longer our patient anymore.’ And it’s like, ‘No, no, no; you can’t do this’. [FNHP 2, Yarn 1]*

*When I do get calls from people, it’s because a patient hasn’t showed up, but they’ve never referred this person to me. But I’m expected to support them. And I keep saying, well, this isn’t an Aboriginal health issue, this is a failure of the service. And I said, what is it that you expect me to do to support you in this space? You’ve not even referred him to me. [FNHP 3, Yarn 7]*

*I said, “At the end of the day, this is a human being you’re talking about. This is a wife, this is a husband, this is a sister, this is somebody,” and not, “Oh, this is a Stage 4 lung cancer patient with metastatic liver disease.” [FNHP 3, Yarn 6]*

*And then education wise for staff, I think it really needs to be drummed into a lot of clinical staff about kinship [...] They didn’t attend their appointment, but there’s other problems within the family. [FNHP 2, Yarn 1]*

FNHPs described how they were able to advocate for the patient in discussions with the clinical team, to ensure that the patient’s preferences, personal circumstances, and culture are considered in the treatment context. They also flagged that they felt that patients are not always properly informed by their treating specialists about the efficacy of treatment, side effects and their prognosis. The way in which information was poorly communicated and not meaningfully understood by patients caused much angst for FNHPs.

*A clinician has a duty of care to their patient…treatment …shouldn’t be forced, they shouldn’t be giving them false hope … thinking that it’s going to prolong their … this is about giving patients the space to make informed decisions around their care. [FNHP 3, Yarn 6]*

*[A patient] said to me, “So what’s my exact diagnosis?” And I said, “Multiple myeloma and it’s very advanced.” So, he said, “How do I spell that?” And he got in and he is doing a Google search and then he is just sitting there quietly for a minute and then he goes, “I don’t have cancer, do I?” And I said, “Yeah, you do. It’s a blood cancer.” And he goes, “Oh,” nobody has told him anything. [FNHP 2, Yarn 6]*

As a trusted support person, FNHPs would often explain and translate much of the Western medicalised language and jargon that patients had difficulty understanding. Sometimes FNHPs were also administering supportive care and psychosocial measures to patients other than the WM2 A, and they often found that patients were confused and troubled by the questions included in these non-First Nations specific measures.

*I genuinely hate doing ESAS and DTs. [….] And I don’t agree with them. I think they’re inflicting more trauma than they’re actually helping them. [FNHP 3, Yarn 4]*

*They identify in the wellness survey because when you’re talking about their worries, their anxiety, it’s, I guess, their whole life and all the issues. Whereas if you go, oh have you got any anxiety? And they’ll be like, ‘What’s anxiety?’ I find that a lot. I have a lot of people who go, “What’s anxiety?” [FNHP 3, 1, Yarn 1]*

#### Incorporating First Nations ways

FNHPs spoke at length about the support they provide for their patients. Appropriate care was said to include cultural elements, acknowledgement of past traumas, and supporting the whole person, not just their cancer. Providing such elements of care manifests in positive well-being experiences of both the patient and the FNHP. FNHPs would often read body language and non-verbal cues of the patient to determine their level of comfort and respond accordingly to help patients feel more relaxed.

*I had [a male patient] sit in the waiting room and I said to my boss, “Can you just move him outside?” She’s like, “What?” And I was like, “You can’t be sitting there with everyone just staring at him; it’s making it worse. Can you please get him outside?” So, we put some chairs outside. As soon as they automatically put the chairs out, he automatically got up and walked out. [FNHP 1, Yarn 1]*

FNHPs were responsive to patient needs, demonstrating deep knowledge of First Nations’ experiences in health care. They were able to provide culturally considered care tailored to patient experiences, which was felt to increase patients’ sense of safety. The foundational cultural bond shared between FNHPs and patients formed the basis of a trusting relationship in a health care context.

*So just trying to be supportive of* [female patient] *when she’s coming in clinic, because she gets a lot of anxiety. And sometimes it’s just me sitting in there, having a yarn with her and talking about mob stuff or whatever. [FNHP 3, Yarn 3]*

*They’ll come to me when—if she needs something they’ll come to me. […] It’s just like when you’re ready this is what we can do. [FNHP 4, Yarn 10]*

FNHPs could understand and relate to the competing priorities experienced by many of their First Nations patients in addition to receiving cancer treatment. FNHPs were able to provide holistic support to patients that respectfully considered these challenges.

*I think, from her point of view, her real priority is just creating memories with her grandson.’Cause he’s only six months old or so.” [FNHP 3, Yarn 2]*

#### Serving your community

FNHPs spoke about the complexity of managing community and cultural obligations to their First Nations patients, the community-controlled health sector, and the First Nations community more broadly. FNHPs were keenly aware of their responsibility to do the best that they could for their community, particularly with regard to ensuring patients received culturally safe and holistic care. FNHPs spoke to their unique position in being connected with their patients—who are members of their own community—in ways that other health professionals may not be. On one hand, this connection gave them greater insight into the experiences of First Nations patients to better meet their healthcare needs. On the other hand, FNHPs found that work and personal lives were not so easily separated.

*It’s one thing to leave work at work, but our work comes home with us, regardless of whether we intentionally leave it there or not… So as a result, our home lives are affected, our relationship with our family gets affected, but also our reputation in community gets affected. [FNHP 3, Yarn 9]*

*It affects the way that I can go down the street and run into people without people accusing me of letting their cousin down. Even though we keep patient confidentiality, I’m the only one in this position, so they know that it’s me. And, yeah, it does take a heavy toll. I have one person come up to me at the shops while I was with my kids, asking me what was happening with such and such and I refused to talk about it. [FNHP 3, Yarn 5]*

*I think [other staff] are seeing the role of the Aboriginal Health Workers differently to what we see it. We’re not doing preferential treatment for anyone, it’s just the way we are as Aboriginal people in cultural ways and all that stuff. [FNHP 4, Yarn 10]*

Whilst pressure was felt on a personal level, the ability to support community members through their cancer journey was also said to be a privilege.

*If they’re not happy with the way that they were treated, and they expect us – that’s the thing, they expect us to do something about it. There’s a level of responsibility that comes with that. [FNHP 3, Yarn 9]*

*And I know, like I said, I’m from this community, so if I muck up, they’re going to tell me. [FNHP 4, Yarn 10]*

*And so, going back to the survey, and you’re sitting there and they’re sharing those stories and you realise, “Oh, I’ve missed out on so much, this is bullshit.” Like, it makes you angry and frustrated, but you also know you’re really in a privileged situation because you’re being exposed to these people who have got these amazing stories and some of them are just downright hilarious and you just think, oh my God, this is so good. Like, I wish I could record this and share this because—and it makes your heart sink because you’re just like, oh my God. [FNHP 3, Yarn 1]*

#### Being everything to everyone

Working in a First Nations-identified role within a hospital was described as incredibly rewarding but also very challenging. Elements of the role, including patient advocacy, working with and sometimes against Western systems, and managing the colonial load and competing demands of the workplace and community, were complex to navigate and took a substantial toll on the FNHPs.

*But it is difficult, it’s a difficult thing. I can honestly say that, in my five months that I’ve been here, I can’t see this being a career in terms of being an Aboriginal Health Worker. It is heavy. [FNHP 3, Yarn 6]*

FNHPs discussed their roles as being less defined than other positions in the healthcare system. Given that these roles are First Nations identified and culturally specific, FNHPs spoke to the experience of being pulled into any issue that involved an identified patient, regardless of whether the FNHP was linked with the client or not.

*You are literally that link that the hospital and community needs. And there’s a lot of weight and pressure that’s attached to that because you know that if you’re putting all that resource into that one person, you’re not going to get a good outcome. [FNHP 3, Yarn 1]*

This issue was felt keenly in the current project, given FNHPs were hired specifically to deliver the WM2 A measure and assist its implementation in cancer services. FNHPs felt that they were often pulled into addressing issues far beyond the scope of their role. Often, they felt that this was to fill gaps in the system around providing care to First Nations patients.

*I’m also keenly aware that there are a lot of gaps in the services, and I don’t feel that using an Aboriginal Health Worker to fill those gaps is probably sustainable in terms of long-term planning. [FNHP 3, Yarn 9]*

FNHPs shared that this often made them feel undervalued in the cancer service, and that little effort was given by staff to understand what the scope of their role was. FNHPs felt that the default for most hospital staff is to direct any issue involving a First Nations patient to them. They described constantly trying to explain the scope of their role to other staff, which they found exhausting and frustrating.

*I’m literally just the wastebasket of Aboriginality, it’s insane. And I spent a lot of my time pushing back, so letting social workers know what my scope of practice is and defining those boundaries and explaining what it is. [FNHP 3, Yarn 5]*

FNHPs spoke about the need for greater clarity and specificity in the roles to cater to the needs of First Nations patients and reduce the burden on the few staff who are currently providing cultural support not only to the patient but also to other health staff.

*I should have been a nurse, then they can all back off and leave me the hell alone because then they won’t bug me about psychosocial stuff that clearly has nothing to do with me, but yet somehow, because they are Aboriginal, it does. [FNHP 3, Yarn 5]*

In addition to being pulled in a variety of directions in their role, FNHPs described carrying a great cultural load by providing cultural support to First Nations patients, families and communities, as well as cultural guidance to their non-First Nations colleagues. They felt a great responsibility to make the health system as culturally safe as possible.

*You hope that you can impart some wisdom and knowledge to the consultants about the way that we approach and support our mob in a more culturally safe way. Because when they’re safe, we’re safe. We can go to work, and we can be happy knowing that we’re in an environment that supports us. [FNHP 3, Yarn 8]*

#### Stigma around the role

The role of FNHPs within the mainstream healthcare system was discussed as not fitting within the Western understandings of what a health professional does. FNHPs spoke to the stigma they felt and the colonial load that existed around their roles and the work they conducted within those roles.

*Our people don’t like being inside, so we go out and have a cuppa and have yarns out in the garden. We talk about our families and how their sickness is hurting them and their families. We makes plans, we share our truth and we draw strength from each other. But the people on the outside just see us being lazy and just sitting around having a yarn and having coffees all day, and then be subjected to comments like oh that must be nice, I wonder how many patient they seen today., like it’s a competition. They don’t actually see the real work that’s actually taking place. [FNHP 3, Yarn 9]*

Cultural elements of care, like Yarning with patients, seeking culturally appropriate spaces to meet with patients outside of the hospital and time spent building rapport and trust with each patient, were not felt to be valued by the broader cancer care team.

*But the same process applies when you’re in the clinic. You walk in, you see Aunt and Uncle and you’re like, what can I do for you? Can I get you a coffee? How are you today? Have you got a social worker? […] you still get in there and you do what you normally do, whether you’re out in community or not. It’s just they think that what we’re doing is preferential treatment, but it’s normal. [FNHP 3, Yarn 7]*

*Oh, they’ve seen me six times today and every time I’ve been with like a different person, we’re sitting down and having a Yarn. But like I said, the information, the important information you can get from having a Yarn, which like you said, like they said, can then be given to the consultants, given to the specialists. [FNHP 4, Yarn 10]*

There was a strong sense that the roles of FNHPs were caught in an irreconcilable dilemma. For while FNHPs were expected to have all the answers and solve all problems facing First Nations patients, they also found that culturally appropriate and trauma-informed approaches to building trust and connections with patients were frowned upon and not understood or respected by colleagues and systems.

*And it’s those connections that they actually rely on us to keep [First Nations patients] in the service long enough, so they can do the treatment, but yet we’re also ridiculed for the very same thing. [FNHP 3, Yarn 9]*

FNHPs described the urgent need for a more integrated and multi-disciplinary approach to providing holistic care for First Nations cancer patients that extends beyond the treatment setting and does not rest solely on First Nations staff.

*But they use you as a scapegoat. ‘Well, you need to fix this.’ I’m like, ‘No, we need to sit down and talk together and see how we can fix this together.’ ‘Cause I don’t want them thinking that, it’s just an Aboriginal problem. Let an Aboriginal person sort it. Like, this is a community effort. And that responsibility shouldn’t be just left on an Aboriginal health worker. And clinicians should be getting involved too and actively engaging in community to support them if they want to close the gap. [FNHP 3, Yarn 7]*

This reflects the sense of frustration and isolation felt by FNHPs as supporters and advocates for First Nations peoples in the health system and underscores the shared responsibility of all staff to work towards addressing the inequities experienced by First Nations peoples and not let this responsibility fall only to FNHPs.

## Discussion

This paper provides valuable insights into the strengths of, and challenges faced by, FNHPs in delivering holistic support to First Nations peoples with cancer. The longitudinal participatory approach used in this study enables an in-depth understanding of the knowledge shared by FNHPs about their experiences as they navigate the intersection of Western medicine and First Nations culture, priorities, and the intergenerational trauma that the has been inflicted on First Nations peoples at the hands of medical professionals.

The six intersecting themes that emerged through our team’s CYM applied to the Yarning Circles with FNHPs are individually not unique. They each align in various ways with research conducted into the provision of healthcare for First Nations people in other conditions and healthcare settings. However, cancer care’s medical complexity, emotional impact, multidisciplinary nature, and focus on prevention and personalized treatment exacerbate the challenges in keeping FNHPs culturally safe whilst providing culturally appropriate and holistic care for First Nations people. The relevance of our findings in guiding improved cancer care for First Nations peoples are discussed and considered below, around five key areas of implication for cancer care delivery.

### Recognising the invaluable role of FNHPs in patient- and family-centred care

The findings of this study highlight the vital contribution that FNHPs can provide in the delivery of cancer care for First Nations peoples that is truly patient- and community-centred. FNHPs in this study created trusted and safe spaces for patients to find some comfort during a highly stressful and difficult time, and to share details of their lives and experiences that impact their health, treatment, and wellbeing. Such a trusting and safe space facilitated improved communication and access to information, which in turn, enabled patients to make informed decisions about their condition, treatment options, rights, and support services. Further, patients were afforded greater agency to determine their engagement with treatment and demanded that health services consider their cultural needs and preferences.

It was evident that the administration of the WM2 A measure by FNHPs with cancer patients created a trusted and safe space for patients to share their feelings, experiences and challenges with staff. Our findings resonate with existing evidence about the critical aspects of cancer care that shape First Nations patients’ experiences, such as feeling safe in the system, the importance of First Nations staff, and effective communication [44]. Similarly, our findings of the invaluable link that FNHPs provide between the health service and First Nations communities have been demonstrated in other health and geographic areas [45–47]. Moreover, the importance of patients’ trust in their health care workers has been reported as a critical factor in culturally safe care [48].

### Balancing the value of and burden on FNHPs

Given the clear benefits of patient-centred and culturally grounded cancer care that FNHPs can deliver, it is unsurprising that our findings highlighted the immense burden on these roles to meet the needs of First Nations patients as well as the expectations of the Western health system. The value of having the WM2 A framework to guide these discussions with cancer patients in the provision of culturally safe care for patients is clear. However, an awareness of the burden on FNHPs of administering the measure, in terms of time and emotional toll, is also important to acknowledge. Ensuring that FNHPs are supported and resourced within mainstream health services to create these safe spaces for patients is critical in retaining FNHPs and in the provision of appropriate and effective cancer care for First Nations peoples. These insights illustrate how and why burnout is so high among First Nations roles, and the challenges of attracting and retaining First Nations staff in health care [17]. The need for greater clarity and delineation around roles, responsibilities and expectations for FNHPs was strongly called for by the knowledge holders in this study. In order for this to be effective, FNHPs roles and responsibilities must be clearly communicated to non-First Nations colleagues in order to quell the practice of First Nations staff being the catch all solution for supporting First Nations patients [45, 47].

### Inclusion of First Nations culture and knowledges in mainstream healthcare

The current study demonstrated the need for FNHPs to forge culturally safe spaces within hospital setting for both patients and staff which added substantial workload and emotional toll on the FNHPs. Constantly fighting against a system that does not respect First Nations culture and knowledge was exhausting and debilitating for FNHPs, particularly when this fight is in addition to the complexities and stresses of providing support to First Nations peoples who are undergoing treatment for cancer. These experiences highlight the exclusion of, and lack of acknowledgement and respect for, First Nations culture in mainstream medical settings, exemplifying the stress that this occasions for staff. [24] While government plans and guidelines spell out the need for First Nations culture and knowledges to be incorporated in mainstream healthcare [13, 49], achieving this requires large scale, sustained efforts to educate Australian health staff, and the Australian public more broadly, about the true history and ongoing impacts of colonisation, as well as the wisdom and strengths of First Nations Australian cultures.

### Eradicating racism and stigma facing FNHPs

The conflicting challenges faced by FNHPs of being a catch-all for everything, while also feeling stigmatised in their roles, again speak to the prevailing systemic and interpersonal racism in the health system, which leads to high rates of burnout for FNHPs. Experiences of racism and stigmatisation in healthcare, for both staff and patients, are not exclusive to cancer care nor to First Nations peoples [15, 50]. Previous studies highlight how mainstream staff construct healthcare as impartial and that racism is not something that is commonly or easily talked about in the workplace [50]. There is an urgent need for racism in healthcare in all its forms to be discussed, highlighted and explored, in order to make any tangible steps towards eradicating its pervasive impacts on First Nations peoples working in healthcare.

### Ensuring policy supports the well-being of patients and staff

An increasing awareness of the important role of FNHPs in cancer care is evident in the inclusion of references to FNHPs in health policy documents, such as the Australian Cancer Plan [19], the National Aboriginal Community Controlled Health Organisation (NACCHO) Aboriginal, and Torres Strait Islander Cancer Plan [20]. However, our findings of the depth and variety of pressures experienced by FNHPs providing culturally supportive cancer care expose an urgent need for action to ensure that FNHPs currently working in cancer care are more effectively supported to retain them and that much-needed new FNHPs are not deterred from working in this space. First Nations people must guide this action to ensure that it truly meets their needs.

### Recommendations stemming from the Knowledge Shared

The knowledge shared by FNHPs in this paper highlights key areas that remain unaddressed to more effectively support First Nations peoples working in Australian health services to provide culturally grounded care for First Nations patients. We put forward the following recommendations in response to the knowledge shared by the FNHPs in this project.

#### Cultural safety training

The ubiquitous experiences of racism and stigma by FNHPs highlight that current approaches to cultural safety training are not having a tangible effect on the ground. Ongoing cultural safety training that is grounded in critical self-reflection to unveil and address unconscious bias, privilege and racism among non-Indigenous health workers has been shown to facilitate a greater inclusion of First Nations culture and knowledge in mainstream healthcare [51]. More effective and holistic approaches to eradicate racism and stigma in healthcare settings must be prioritised urgently responded to. Moreover, engaging with patients and First Nations communities in culturally sensitive and appropriate ways should be the responsibility of all staff and clinicians, which must be grounded in deeper understandings and respect for First Nations culture and the processes of colonisation. However, it is also recognised that clinicians are often not adequately supported by the health system to engage effectively in culturally safe care.

Currently, mandatory cultural training is limited to annual online modules, with face-to-face training offered infrequently and inconsistently. This is insufficient given the nature of clinical roles, where high workloads and competing demands affect memory retention and sustained learning. The issue is not that clinicians are uninvolved in care, but that their engagement is often not culturally appropriate or grounded in a deeper understanding of First Nations cultures and the ongoing impacts of colonisation. To address this, structural transformation of the colonial health system is essential to better support all staff—clinicians and non-clinicians alike—in engaging meaningfully with First Nations peoples.

Health departments should prioritise the delivery of cultural safety programs at the frontline of care and appoint on-the-ground cultural safety champions to support continuous, embedded practice change. One example of such change is the provision of protected time for all health staff to participate in culturally significant events such as NAIDOC week. This would create opportunities for reflection, connection, and strengthened commitment to culturally safe practice.

#### Education and management

There is an essential need for all staff and management within mainstream cancer services to be educated about the critical value of FNHPs in providing effective patient-centred care. Accordingly, FNHPs require clearer, more delineated roles and responsibilities to avoid FNPHs being inundated as the ‘catch all’ support person for First Nations cancer patients. This would help to clarify which areas of responsibility are appropriate for FNHPs to assume and which roles must be assumed by other health and clinical staff. This is critical to avoid FNHPs experiencing over-burden, burnout, and colonial load.

#### Policy and advocacy

Embedding policies and guidelines such as the National Aboriginal and Torres Strait Islander Health Workforce Strategic Framework and Implementation Plan 2021–2031 [52], Optimal Care Pathway for Aboriginal and Torres Strait Islander people with cancer [13] and the Australian Cancer Plan [49] needs to be a priority in cancer services. Achieving equity in cancer outcomes for Aboriginal and Torres Strait Islander peoples is a key aim of these documents. Targeted recruitment and retention strategies, such as scholarships and career pathways, are essential to encouraging First Nations individuals to pursue careers in cancer care. Equally important is ensuring that FNHPs are not only recruited but culturally and professionally supported to deliver safe, patient centred care. To measure the impact of these policies and ensure they are responsive to community needs, data-driven accountability and surveillance systems must be established and made mandatory. This includes embedding the Maiam Nayri Wingara principles for data sovereignty, ensuring First Nations people have control over the collection, access and use of their health data.

These efforts can help create a more inclusive and supportive environment for FNHPs, ultimately improving health outcomes for First Nations communities, whether they are using or delivering health services.

#### Strengths and limitations

The strength of this research was the participatory approach used which privileged FNHPs voices and facilitated their participation in the study and involvement in the interpretation and guiding the analysis. This approach is consistent with Rigney’s Indigenist research approach. [33] Furthermore, this project was co-led and guided by a majority First Nations research team. Our approach to knowledge creation upholds First Nations Australians’ right to control and autonomy over health and wellbeing outcomes in the context of colonisation and its ongoing effects.

This paper forms part of a larger study designed to focus on measuring the well-being of First Nations patients using mainstream cancer services; it was not designed to understand the experiences of FNHP employees who applied the measure. As such, while the perspectives of Knowledge Holders collected are in-depth and long-term, the sample size was small. This characteristic has implications for the generalisability of our findings to other settings and jurisdictions. While our findings align strongly with previous research in other contexts, this study provides a foundation for further research to explore and substantiate the experiences of FNHPs in other states and health care settings.

## Conclusion

This study offers a timely insight into the realities facing First Nations peoples working in cancer care with the hope of guiding changes that will reverse the current trends of FNHP burnout and exit from the profession. The experiences of the FNHPs in this study highlight the need for mainstream cancer services to recognise the value of FNHPs in patient-centred care; balance this value with the burden on FNHPs; foster greater inclusion of First Nations culture and knowledge in mainstream healthcare; and actively focus on addressing racism and stigma facing FNHPs. In doing so, we create opportunities to facilitate equitable access to culturally safe cancer knowledge, treatment, services, and support among First Nations peoples and genuinely work towards closing the gap.

## Data Availability

The transcripts that form the data of this project are not available to the public.

## Appendix 1. Yarning circle guide

1. STAFF VIEWS ABOUT THE MEASURE
  - How did you feel about applying the measure to patients?
  - How have patients been receiving the measure?
  - What do patients say about the measure in comparison to non-Indigenous measures?
2. WHAT WORKS
  - What works for you in implementing the measure?
3. CHALLENGES
  - Have you experienced any challenges implementing the measure?
  - Would you change anything about the measure?
4. REFERRAL PATHWAYS
  - Have you referred any patients for low scoring items?
5. ANY OTHER INTERESTING INSIGHTS

## References

[1] Australian Institute of Aboriginal and Torres Strait Islander Studies (2023) Australia’s first people. Available from: https://aiatsis.gov.au/explore/australias-first-peoples. Accessed 1/10/2024

[2] Australian Institute of Health and Welfare (2024) Aboriginal and Torres Strait Islander Health Performance Framework: summary report August 2024. AIHW: Australian Government

[3] Australian Institute of Health and Welfare (2024) Aboriginal and Torres Strait Islander health performance framework—summary report—life expectancy. Available from: https://www.indigenoushpf.gov.au/report-overview/overview/summary-report/4-tier-1-%E2%80%93-health-status-and-outcomes/life-expectancy. Accessed 1/10/2024

[4] Cunningham J et al (2008) Incidence, aetiology, and outcomes of cancer in Indigenous peoples in Australia. Lancet Oncol 9(6):585–595 18510990 10.1016/S1470-2045(08)70150-5

[5] Condon JR, Barnes T, Cunningham J, Armstrong BK (2004) Long-term trends in cancer mortality for Indigenous Australians in the Northern Territory. Med J Aust 180(10):504–507. 10.5694/j.1326-5377.2004.tb06052.x 10.5694/j.1326-5377.2004.tb06052.x 15139826

[6] Valery PC et al (2006) Cancer diagnosis, treatment, and survival in Indigenous and non-Indigenous Australians: a matched cohort study. Lancet 367(9525):1842–1848 16753487 10.1016/S0140-6736(06)68806-5

[7] Paterson C et al (2024) Understanding the needs and preferences for cancer care among First Nations people: an integrative review. J Adv Nurs 80(5):1776–812 38018290 10.1111/jan.15968

[8] Shahid S, Finn LD, Thompson SC (2009) Barriers to participation of Aboriginal people in cancer care: communication in the hospital setting. Med J Aust 190(10):574–579 19450207 10.5694/j.1326-5377.2009.tb02569.x

[9] Shahid S et al (2016) Factors contributing to delayed diagnosis of cancer among Aboriginal people in Australia: a qualitative study. BMJ Open 6(6):e010909 27259526 10.1136/bmjopen-2015-010909 PMC4893856

[10] Carson BE, Bailie RS (2004) National health workforce in discrete Indigenous communities. Aust N Z J Public Health 28(3):235–245 15707170 10.1111/j.1467-842x.2004.tb00702.x

[11] Hill S et al (2010) Survival disparities in Indigenous and non-Indigenous New Zealanders with colon cancer: the role of patient comorbidity, treatment and health service factors. J Epidemiol Commun Health 64(2):117–123 10.1136/jech.2008.083816 20056966

[12] Gracey M, King M (2009) Indigenous health part 1: determinants and disease patterns. Lancet 374(9683):65–75 19577695 10.1016/S0140-6736(09)60914-4

[13] Cancer Australia (2024) Optimal care pathway for Aboriginal and Torres Strait Islander people with cancer, 2nd edn. Cancer Australia, Surry Hills

[14] Sanjida S et al (2022) Indigenous Australians’ experiences of cancer care: a narrative literature review. Int J Environ Res Public Health 19(24):16947 36554828 10.3390/ijerph192416947 PMC9779788

[15] Bailey J, Blignault I, Carriage C, Demasi K, Joseph T, Kelleher K, Lew Fatt E, Meyer L, Naden P, Nathan S, Newman J, Renata P, Ridoutt L, Stanford D, Williams M (2020) ‘We Are working for our people’: growing and strengthening the Aboriginal and Torres Strait Islander health workforce. Career Pathways Project Report. The Lowitja Institute, Melbourne

[16] Australian Institute of Health and Welfare (2023) Cultural safety in health care for Indigenous Australians: monitoring framework. Available from: https://www.aihw.gov.au/reports/Indigenous-australians/cultural-safety-health-care-framework/contents/summary. Accessed 1/10/2024

[17] Australian Institute of Health and Welfare ((2024)) 3.12 Aboriginal and Torres Strait Islander people in the health workforce. Available from: https://www.Indigenoushpf.gov.au/measures/3-12-atsi-people-health-workforce#:~:text=For%20Commonwealth-funded%20Indigenous%20primary%20health%20care%20organisations%2C%20Indigenous,Indigenous%20staff%2C%204%2C208%20%2894%25%29%20were%20employed%20at%20ACCHSs. Accessed 1/10/2024

[18] Health Workforce Australia (2011) Growing our future: the Aboriginal and Torres Strait Islander health worker project final report. Health Workforce Australia, Adelaide

[19] Australia, C (2023) Australian Cancer Plan. Available from: https://www.australiancancerplan.gov.au. Accessed 1/10/2024

[20] National Aboriginal Community Controlled Health Organisation (2023) Aboriginal and Torres Strait Islander Cancer Plan. NACCHO, Canberra

[21] Conway J, Tsourtos G, Lawn S (2017) The barriers and facilitators that Indigenous health workers experience in their workplace and communities in providing self-management support: a multiple case study. BMC Health Serv Res 17(1):319 28468612 10.1186/s12913-017-2265-5 PMC5415721

[22] Cosgrave C, Maple M, Hussain R (2017) Factors affecting job satisfaction of Aboriginal mental health workers working in community mental health in rural and remote New South Wales. Aust Health Rev 41(6):707–711 27914487 10.1071/AH16128

[23] Topp SM, Edelman A, Taylor S (2018) “We are everything to everyone”: a systematic review of factors influencing the accountability relationships of Aboriginal and Torres Strait Islander health workers (AHWs) in the Australian health system. Int J Equity Health 17(1):67 29848331 10.1186/s12939-018-0779-z PMC5977558

[24] Gatwiri K, Rotumah D, Rix E (2021) BlackLivesMatter in Healthcare: racism and Implications for health inequity among Aboriginal and Torres Strait Islander peoples in Australia. Int J Environ Res Public Health 18(9):4399 33919080 10.3390/ijerph18094399 PMC8122304

[25] Meyer L, Joseph T, Anderson-Smith B, Blignault I, Demasi K, Lew Fatt E, Nathan S (2020) Career pathways for the Aboriginal and Torres Strait Islander Health Workforce: literature review report. Career Pathways Project. The Lowitja Institute, Melbourne

[26] Cotter P et al (2022) Coping with the emotional impact of working in cancer care: the importance of team working and collective processing. Front Psychol 13:877938 35911049 10.3389/fpsyg.2022.877938 PMC9336679

[27] Bernardes CM et al (2018) Lessons learned from a pilot study of an Indigenous patient navigator intervention in Queensland, Australia. Eur J Cancer Care (Engl) 27:27(1) 10.1111/ecc.12714 28513056

[28] Taylor EV et al (2020) “We’re very much part of the team here”: a culture of respect for Indigenous health workforce transforms Indigenous health care. PLoS ONE 15(9):e0239207 32960933 10.1371/journal.pone.0239207 PMC7508383

[29] Zubrzycki J, Shipp R, Jones V (2017) Knowing, being, and doing: Aboriginal and non-Aboriginal collaboration in cancer services. Qual Health Res 27(9):1316–1329 28682709 10.1177/1049732316686750 PMC5502907

[30] Howard K et al (2020) What matters 2 adults: a study protocol to develop a new preference-based wellbeing measure with Aboriginal and Torres Strait Islander adults (WM2Adults). BMC Public Health 20(1):1739 33203391 10.1186/s12889-020-09821-z PMC7672853

[31] Howard K et al (2024) Development of the What matters 2 adults (WM2A) wellbeing measure for Aboriginal and Torres Strait Islander adults. Soc Sci Med 347:116694 38569315 10.1016/j.socscimed.2024.116694

[32] Given L (2008) Participants as co-researchers. In: The SAGE encyclopedia of qualitative research methods. SAGE Publications, Inc., pp 600–601. 10.4135/9781412963909.n310

[33] Rigney L-I (1999) Internationalization of an Indigenous anticolonial cultural critique of research methodologies: a guide to indigenist research methodology and its principles. Wicazo Sa Review 14(2):109–121

[34] Fitzsimmons T, Yates MS, Jordan R, Callan VJ (2024) Co-creating impact: positioning Indigenous knowledge holders as expert researchers. Equal Divers Incl. 10.1108/edi-09-2023-0315

[35] Alvesson M, Sköldberg K (2018) Reflexive methodology, vol 1–0. SAGE Publications Ltd. 10.4135/9781036211523

[36] Harvey G, Kitson A (2016) PARIHS revisited: from heuristic to integrated framework for the successful implementation of knowledge into practice. Implement Sci 11(1):33 27013464 10.1186/s13012-016-0398-2 PMC4807546

[37] Bessarab D, Ng’Andu B (2010) Yarning about yarning as a legitimate method in Indigenous research. Int J Crit Indig Stud 3(1):37–50

[38] National Health and Medical Research Council (2018) Ethical conduct in research with Aboriginal and Torres Strait Islander Peoples and communities: guidelines for researchers and stakeholders. Commonwealth of Australia, Canberra

[39] Shay M (2021) Extending the yarning yarn: Collaborative Yarning methodology for ethical Indigenist education research. Aust J Indig Educ 50(1):62–70

[40] Garvey G et al (2021) The fabric of Aboriginal and Torres Strait Islander wellbeing: a conceptual model. Int J Environ Res Public Health 18(15):7745 34360037 10.3390/ijerph18157745 PMC8345714

[41] Garvey G et al (2024) What matters to Aboriginal and Torres Strait Islander youth (WM2Y): a study protocol to develop a national youth well-being measure. BMJ Open 14(3):e076119 38508611 10.1136/bmjopen-2023-076119 PMC10952880

[42] Lumivero (2023) NVivo (Version 14). www.lumivero.com

[43] Byrne D (2022) A worked example of Braun and Clarke’s approach to reflexive thematic analysis. Qual Quant 56(3):1391–1412

[44] Green M et al (2018) Understanding Indigenous Australians’ experiences of cancer care: stakeholders’ views on what to measure and how to measure it. BMC Health Serv Res 18(1):982 30567564 10.1186/s12913-018-3780-8 PMC6299947

[45] Harfield S et al (2021) Building Indigenous health workforce capacity and capability through leadership - the Miwatj health leadership model. Prim Health Care Res Dev 22:e52 34615567 10.1017/S1463423621000554 PMC8515491

[46] Harfield SG et al (2018) Characteristics of Indigenous primary health care service delivery models: a systematic scoping review. Global Health 14(1):12 29368657 10.1186/s12992-018-0332-2 PMC5784701

[47] Mercer C et al (2012) The experiences of Aboriginal health workers and non Aboriginal health professionals working collaboratively in the delivery of health care to Aboriginal Australians: a systematic review of qualitative evidence. JBI Libr Syst Rev 10(56 Suppl):1–12 27820297 10.11124/01938924-201210561-00026

[48] De Zilva S et al (2021) Culturally safe health care practice for Indigenous peoples in Australia: A systematic meta-ethnographic review. J Health Serv Res Policy 27(1):74–84 34875923 10.1177/13558196211041835

[49] Cancer Australia (2023) Australian Cancer Plan (Summary). Cancer Australia, Surry Hills, NSW

[50] Hamed S et al (2022) Racism in healthcare: a scoping review. BMC Public Health 22(1):988 35578322 10.1186/s12889-022-13122-y PMC9112453

[51] Hall K et al (2023) Evolving beyond antiracism: reflections on the experience of developing a cultural safety curriculum in a tertiary education setting. Nurs Inq 30(1):e12524 36083828 10.1111/nin.12524 PMC10078494

[52] (2022) Australian Government, National Aboriginal and Torres Strait Islander Health Workforce Strategic Framework and Implementation Plan 2021–2031. Department of Health and Aged Care: Canberra

